# Hypothalamic-Specific Manipulation of *Fto*, the Ortholog of the Human Obesity Gene *FTO*, Affects Food Intake in Rats

**DOI:** 10.1371/journal.pone.0008771

**Published:** 2010-01-19

**Authors:** Yi-Chun Loraine Tung, Eduard Ayuso, Xiaoye Shan, Fatima Bosch, Stephen O'Rahilly, Anthony P. Coll, Giles S. H. Yeo

**Affiliations:** 1 University of Cambridge Metabolic Research Laboratories, Institute of Metabolic Science, Addenbrooke's Treatment Centre, Addenbrooke's Hospital, Cambridge, United Kingdom; 2 Center of Animal Biotechnology and Gene Therapy and Department of Biochemistry and Molecular Biology, School of Veterinary Medicine, Universitat Autònoma de Barcelona, Barcelona, Spain; 3 CIBER de Diabetes y Enfermedades Metabólicas Asociadas (CIBERDEM), Barcelona, Spain; University of Parma, Italy

## Abstract

Sequence variants in the first intron of *FTO* are strongly associated with human obesity and human carriers of the risk alleles show evidence for increased appetite and food intake. Mice globally lacking *Fto* display a complex phenotype characterised by both increased energy expenditure and increased food intake. The site of action of FTO on energy balance is unclear. Fasting reduces levels of *Fto* mRNA in the arcuate nucleus (ARC) of the hypothalamus, a site where *Fto* expression is particularly high. In this study, we have extended this nutritional link by demonstrating that consumption of a high fat diet (45%) results in a 2.5 fold increase in Arc *Fto* expression. We have further explored the role of hypothalamic Fto in the control of food intake by using stereotactic injections coupled with AAV technology to bi-directionally modulate Fto expression. An over expression of Fto protein by 2.5-fold in the ARC results in a 14% decrease in average daily food intake in the first week. In contrast, knocking down Arc *Fto* expression by 40% increases food intake by 16%. mRNA levels of *Agrp*, *Pomc* and *Npy*, ARC-expressed genes classically associated with the control of food intake, were not affected by the manipulation of Fto expression. However, over expression of Fto resulted in a 4-fold increase in the mRNA levels of *Stat3*, a signalling molecule critical for leptin receptor signalling, suggesting a possible candidate for the mediation of Fto's actions. These data provide further support for the notion that FTO itself can influence key components of energy balance, and is therefore a strong candidate for the mediation of the robust association between *FTO* intronic variants and adiposity. Importantly, this provide the first indication that selective alteration of FTO levels in the hypothalamus can influence food intake, a finding consistent with the reported effects of FTO alleles on appetite and food intake in man.

## Introduction

Mutations leading to highly penetrant forms of human obesity frequently disrupt the central control of appetite and lead to increased food intake [1]. It will be important to establish whether common polymorphisms associated with obesity act similarly. Common variants within intron 1 of the fat mass and obesity associated gene (*FTO*) are strongly and consistently associated with human adiposity [2], [3]. Ten subsequent studies have examined the influence of *FTO* variants on measures of appetite, food intake or energy expenditure [4]–[13]. While the obesity risk alleles are not associated with reduced energy expenditure, nine of these studies reported an association with increased appetite or measured *ad libitum* food intake [4], [6]–[13]. As yet, there is no evidence that the risk alleles influence *FTO* expression and the variants could conceivably influence adiposity through effects on more distant genes. The global ablation of *Fto* expression in mice [14] results in high early mortality but the surviving mice are small, with reduced lean and fat mass. The absolute food intake of Fto +/+ and Fto −/− mice is identical but when corrected for lean mass, Fto −/− have an increased food intake. Similarly Fto −/− mice have increased oxygen consumption when corrected for lean mass. More recent findings reported in a mouse with a missense loss of function ENU induced mutation in *Fto* [15] strongly suggest that FTO is likely to be involved in the control of energy balance.

*FTO* is widely expressed across multiple tissues, but is most highly expressed in brain and especially the hypothalamus [2], [16]. We have previously shown that murine *Fto* mRNA levels are decreased by fasting and increased by feeding in the arcuate nucleus (ARC) of the hypothalamus [16]. In this study, we have extended this observation by demonstrating that consumption of a high fat diet results in an increase in ARC *Fto* expression. We then examined whether manipulating Fto levels in the ARC might have any effects on spontaneous food intake in rats. In parallel, we examined the effects of manipulating Fto levels in the nearby paraventricular nucleus (PVN), a site known to be important for the control of energy balance but where *Fto* expression was not altered by nutritional state [16]. We demonstrate that manipulation of Fto expression in the arcuate nucleus bi-directionally influences acute food intake in rats, implicating the brain and specifically the ARC in mediating at least some of the effects of FTO on energy balance.

## Results

### Endogenous *Fto* Expression in the ARC

**Figure 1:**
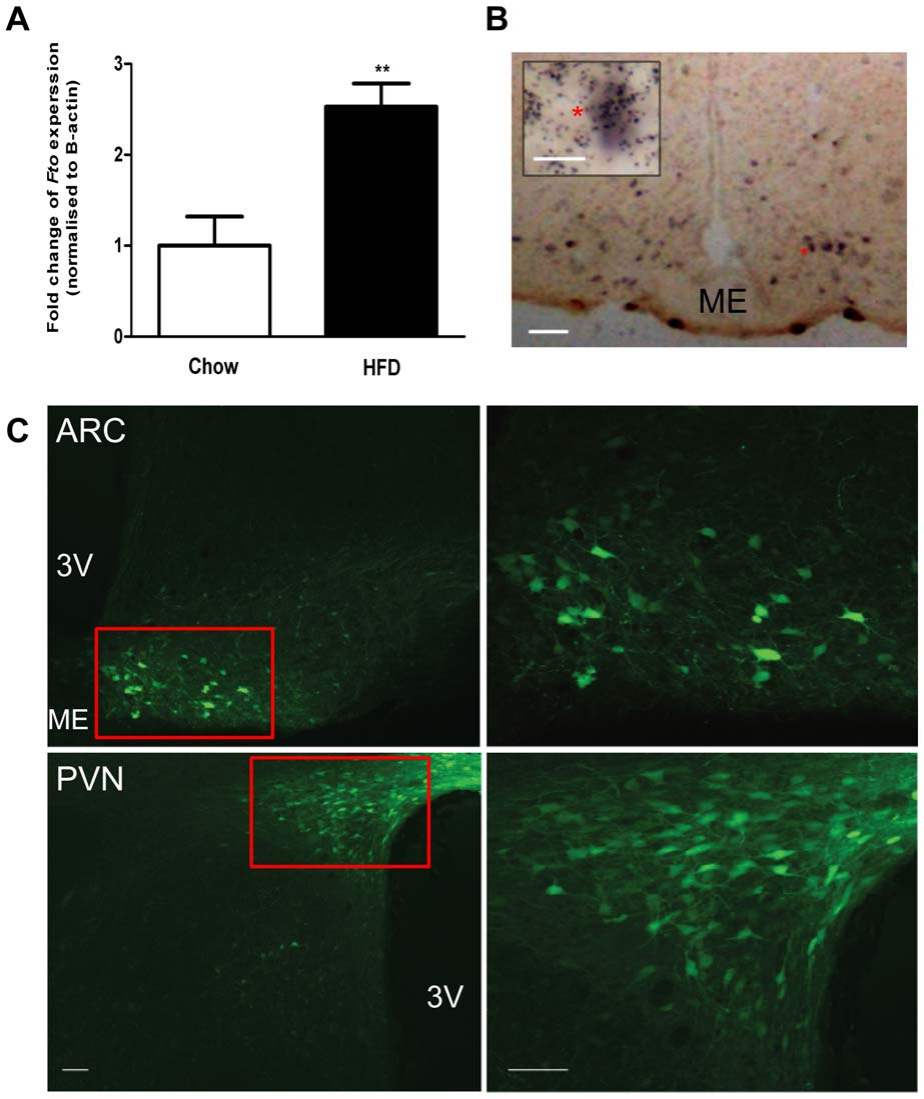
Endogenous *Fto* expression in the ARC. (**A**) *Fto* expression, quantified by real-time RT-PCR, is up-regulated in the arcuate nucleus following a high-fat diet demonstrated by the relative arcuate *Fto* mRNA expression in rat either fed on a standard chow or on a high-fat diet for 10 weeks. Data is represented as the mean±S.E.M of at least 6 independent rats per group; **P<0.01. (**B**) *Fto* mRNA is diffusely expressed throughout the arcuate nucleus and overlaps with *Pomc* neurons. Double *in situ* hybridization detecting *Fto* (^35^S labeled) and *Pomc* (DIG labeled) mRNA in the cells of the arcuate nucleus. Insert in figure shows a high-magnification (scale bar, 20 µm) of a *Pomc* containing neurons colocalised with Fto (marked with *). (**C**) Intra-nuclei bilateral injections of adeno-associated virus (AAV2/7) mediated transfer of GFP cDNA precisely targets the hypothalamic arcuate (ARC) and paraventricular (PVN) nuclei; as demonstrated by photomicrographs of representative coronal sections showing localization of GFP 7 days after injection; right panels, higher magnification of area indicated by red box. Scale bar, 100µm. 3v: third ventricle; ME: median eminence.

We have previously reported that *Fto* expression in the ARC is decreased with nutritional deprivation. We examined the effect of high fat feeding on the expression of *Fto* in hypothalamic nuclei. After 10 weeks of exposure to a high fat (45%) diet, body weight (HFD 549.5±22.6g *vs.* chow 426.8±33.3g; p = 0.01) and fat mass (retroperitoneal fat: HFD 1.3±0.2 *vs.* chow 0.7±0.1% of body weight; p = 0.03) were significantly increased in the high fat fed group without an increase in energy intake (HFD 99±3.7 *vs.* chow 106±2.7Kcal). These physiological changes was accompanied by an increase in the *Fto* mRNA levels in the ARC by 2.5 fold compared to rats provided with regular chow (Fig 1A). In order to determine whether *Fto* is co-expressed with any specific ARC neuronal population, we performed co-localisation studies of *Fto* with the classical ARC expressed anorexigenic pro-opiomelanocortin (*Pomc*) using double *in situ* hybridisation. We confirmed abundant expression of *Fto* in the ARC, and although its expression overlaps with that of *Pomc* (Fig 1B), *Fto* appears to be diffusely expressed throughout the ARC and is therefore likely to be present in many neuronal and non-neuronal populations. Thus, rather than manipulating Fto expression in specific ARC neuronal populations, we chose a strategy employing AAV mediated transfer of Fto cDNA and shRNA coupled with stereotactic injection in order to manipulate Fto expression within the ARC (Fig 1C).

### Manipulation of Fto Expression within the ARC and PVN

In order to determine the optimum AAV serotype and titre, as well as to confirm the accuracy of intranuclear injections, AAV-2/5,2/7,2/8 and 2/9 with GFP were initially produced. AAV-2/7 provided most effective and specific transduction for the hypothalamic nuclei (Fig 1C) and the time course for GFP expression was similar to that reported for other AAV serotypes reported such that the protein was expressed by day 3 following injection and lasted for at least 2 months. Thus all the experimental AAVs used for the following studies were generated using serotype 2/7.

**Figure 2:**
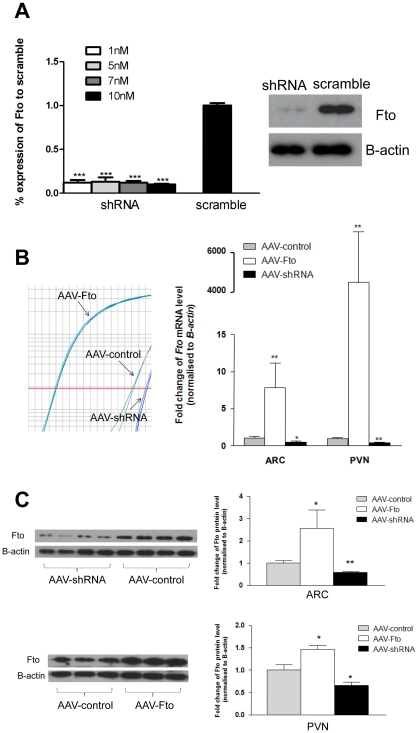
Manipulation of Fto expression within the ARC and PVN. (**A**) Knockdown of *Fto* in GT1-7 cells. *Fto* short hairpin RNA expressed from U6 promoter were transfected into cultured hypothalamic GT1-7 cells resulting in efficient knockdown of *Fto* mRNA (quantified by real time RT-PCR, left panel) and protein (western blot analysis, right panel) compare with cells transfected with scramble shRNA sequence. (**B–C**) Confirmation of AAV mediated transfer of full length cDNA (AAV-Fto) for over expression and shRNA (AAV-shRNA) for knockdown of *Fto* 3 weeks following intra-nuclei injection. Fto expression is increased by AAV-Fto and decreased by AAV-shRNA as compared to controls (empty vector for AAV-Fto and scrambled sequence for AAV-shRNA). Bar graphs show the quantified change in expression of Fto. Response is expressed in terms of fold induction over the control. (**B**) *Fto* mRNA relative to expression of *B-actin* was determined by real-time quantitative PCR. Left panel shows representative amplification curve from PVN micro-punches of AAV-Fto and AAV-shRNA injected rats. (**C**) Fto protein levels were measured by semi-quantitative western blot analysis. Left panel shows representative blots from micro-punched AAV-shRNA injected ARC and from AAV-Fto injected PVN. Western blots were probed with mouse monoclonal antibodies directed against the C-terminal Fto. B-actin was used as a loading control and bands were visualized and semi-quantified using Chemiluminescence. P-value was calculated using a two-tailed distribution unpaired Student's t-test. Data is represented as the mean ±S.E.M of at least 6 independent rats per group. *p<0.05; **p<0.01; ***p<0.001.

To modulate *Fto* expression levels in the ARC and PVN, we generated four AAV 2/7vectors: i.) AAV mediated transfer of full length *Fto* cDNA was used for overexpression; ii.) shRNA targeting *Fto* mRNA for knockdown of gene expression and iii.) two different AAV-controls (empty vector for AAV-Fto and containing a scrambled shRNA sequence for AAV-shRNA). The rat *Fto* specific shRNA that we generated effectively inhibited the expression of endogenous Fto in hypothalamic GT1-7 cells by 90%, whereas transfection with scrambled control did not affect the production of the Fto protein (Fig 2A).

20×10^12^ vg/ml of AAV-Fto, AAV-shRNA and their respective controls were stereotactically injected bilaterally to transduce either the ARC or PVN (Fig 1C) of rats. To assess the specificity of AAV delivery, at the end of the study, 3 weeks after AAV injections, micro-punches from both the ARC or PVN were obtained for all experimental animal and tested for changes in *Fto* expression using real-time quantitative RT-PCR (Fig 2B). From the same dissections, protein expression was measured by semi-quantitative western blotting analysis (Fig 2C). For the over expression studies, at the protein level, we achieved approximately 2.5 fold increase in total Fto levels in the ARC and a 1.5 fold increase in the PVN. Whilst for the knockdown experiments, Fto expression was decreased by 40% in both the ARC and the PVN compared to rats injected with scrambled AAV.

### Effects of AAV-Fto and AAV-shRNA Microinjection in Hypothalamic ARC

**Figure 3:**
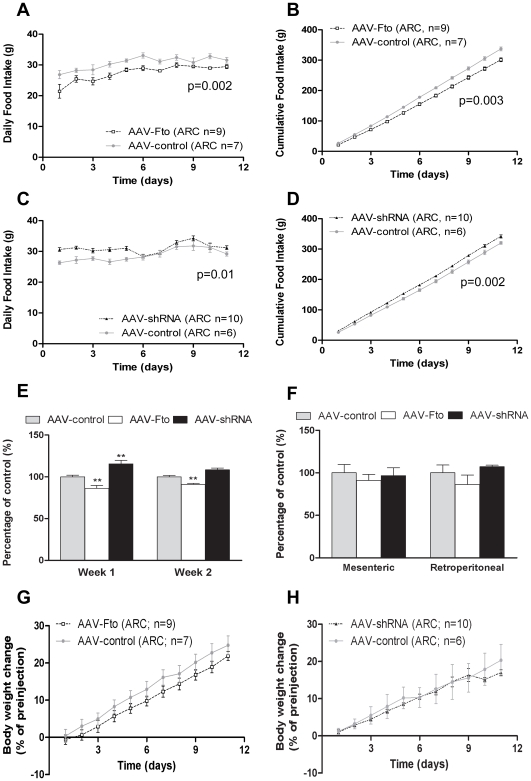
Effects of AAV-shRNA and AAV-Fto microinjection in hypothalamic ARC. Effects of manipulating Fto expression in rat hypothalamic ARC. The 2-way repeated measures ANOVA indicated that ARC injection of (**A**) AAV-Fto significantly reduces daily food intake (F_1,140_ = 15.28; p = 0.002) and (**B**) 2 weeks cumulative food intake (F_1,140_ = 13.26; p = 0.003). Whilst (**C,D**) AAV-shRNA had a significant effect on induction of food intake (daily: F_1,140_ = 8.823; p = 0.01; cumulative: F_1,140_ = 13.73; p = 0.002). (**E**) Significant effects of Fto over expression on food intake last for 2 weeks whilst the effect of Fto knockdown was only significant in the first week. All values are expressed as mean±S.E.M. Statistical comparison between control and treated groups for each site were performed by two-tailed unpaired Student's t-test; **p<0.01.There were no significant effects of ARC Fto over expression and knockdown on (**F**) fat mass normalized to final body weight or (**G–H**) body weight gain expressed as percentage change from pre-surgical weight.

We investigated the effect of Fto on feeding behaviour in rats. At the end of the experiment, we check for Fto expression as an indication for correct site of injection. Three animals (2 from the ARC study and 1 from the PVN study) were excluded from further analysis as expression of both *Fto* RNA and protein levels in these animals were not altered at least 5% as compared to controls. Over expression of Fto in the ARC resulted in a decrease in food intake, compared to rats injected with empty vector (Fig 3A, B). Conversely, a 40% reduction in Fto protein expression resulted in an increase in food intake (Fig 3C, D). Expressing the data as average daily food intake, the peak of the effect was reached within the first week: 14% reduction in the Fto over expressing group and 16% increase in food intake with knockdown (Fig 3E). However, despite having an impact on food intake, there was no significant difference between the body weight of rats that received injection of the AAV-Fto and AAV-shRNA with respective controls (Fig 3G, H). There was also no significant difference in fat mass (Fig 3F).

### Effects of AAV-Fto and AAV-shRNA Microinjection PVN

**Figure 4:**
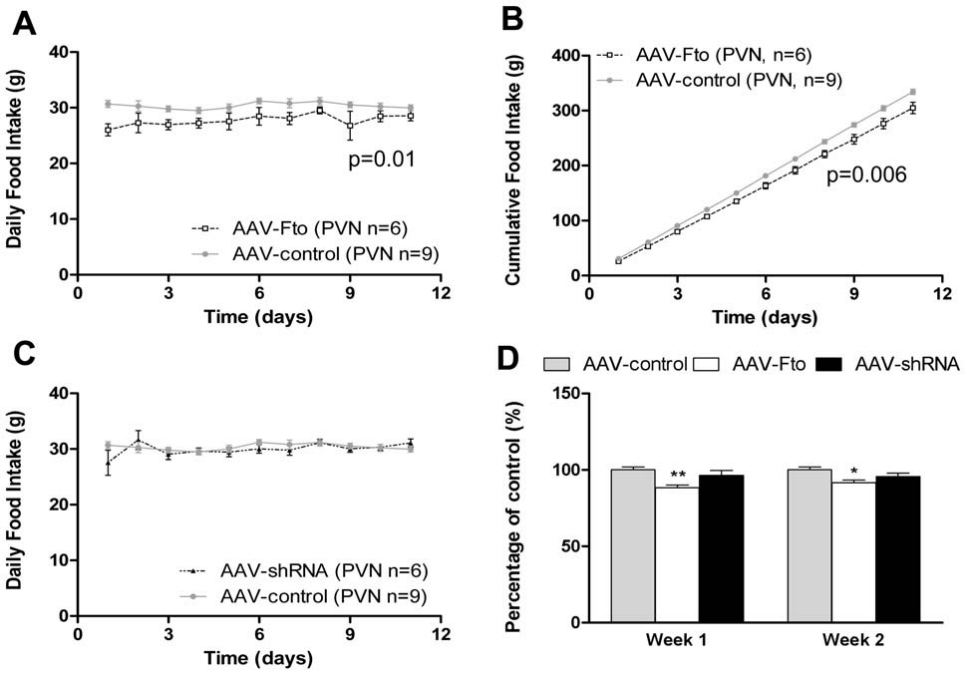
Effects of AAV-shRNA and AAV-Fto microinjection in hypothalamic PVN. Effects of manipulating Fto expression in rat hypothalamic PVN. PVN injection of AAV-Fto reduces (**A**) daily (F_1,130_ = 7.986; p = 0.01) and (**B**) cumulative (F_1,130_ = 10.70; p = 0.006) food intake while (**C**) AAV-shRNA has no impact. (**D**) Food reducing effects of Fto over expression in the PVN was significant for 2 weeks. All values are expressed as mean ±S.E.M. Statistical comparison between control and treated groups for each site were performed by two-tailed unpaired Student's t-test. *p<0.05; **p<0.01. For the analysis of food intake over time, two-way repeated measure ANOVA was used with time and treatment as variables for comparison.

As a comparator, we also perturbed expression of Fto in the PVN. Interestingly, although over-expression of Fto in the PVN also resulted in a reduction of food intake (Fig 4A, B, D), a similar degree of Fto knockdown in the PVN had no effect on food intake (Fig 4C, 4D).

### Effects of Manipulating ARC Fto on Genes Involved in Energy Homeostasis

**Figure 5:**
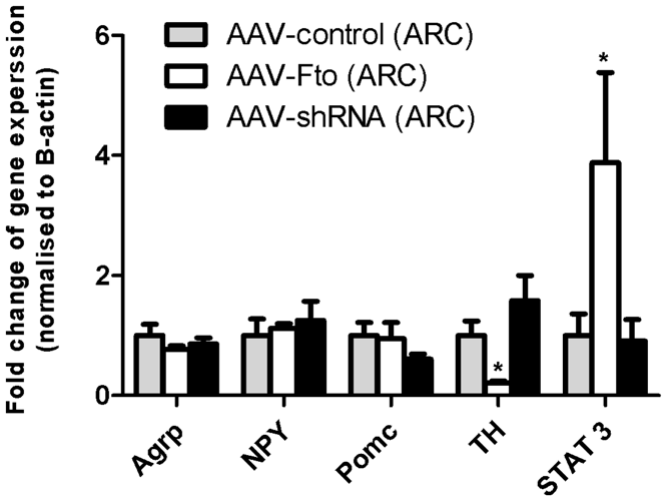
Effects of manipulating ARC Fto on genes involved in energy homeostasis. Bar graphs show no change of *Agrp*, *Npy* and *Pomc* expression in response to arcuate injection of AAV-Fto or AAV-shRNA, while *TH* expression in the ARC decreases by 5- fold and *Stat3* expression increases by 4-fold in response to Fto overexpression. Response of each gene was measured 3 weeks following intra-ARC injection, data normalized to *B-actin* and expressed in terms of fold induction over its expression in controls. P-value was calculated using a two-tailed distribution unpaired Student's t-test. Data is represented as the mean ±S.E.M of at least 6 independent rats per group; *p<0.05.

Using real-time quantitative RT-PCR we determined that the ARC-expressed genes classically associated with the control of food intake, namely, *Agrp*, *NPY* and *Pomc* were not affected by altered Fto expression (Fig 5). However, a 4-fold increase in the expression of the transcription factor *Stat3*, and a 5- fold reduction in *Tyrosine Hydroxylase*, was observed with Fto over expression in the ARC, suggest possible candidates for the mediation of Fto's action.

## Discussion

Previously, we have demonstrated that *Fto* is highly expressed in hypothalamic nuclei critical for regulating energy homeostasis, and that its expression within the ARC is reduced following a 48hr fast. Here we report that a high fat diet results in an increase of ARC *Fto* expression, demonstrating that *Fto* levels can be bi-directionally regulated depending on nutritional status. Due to the diffuse nature of *Fto* expression within the ARC, we chose to manipulate Fto expression throughout the nuclei resulting in a bidirectional impact on food intake.

Lack of FTO from conception, in both humans [17] and mice [14], results in severe growth retardation and high levels of early mortality, accompanied in humans, but not mice, by multiple developmental anomalies. We have reported that FTO is a nuclear-localised 2-oxoglutarate and Fe^2+^ dependent dioxygenase which is capable of demethylating 3-methyl thymine in nucleic acids [16] and that heterozygous, loss-of-function mutations in human *FTO* exist but are found in both lean and obese subjects [18]. Thus, FTO's true, physiological substrate(s) and function remains unclear.

However, evidence up-to-date suggests that the FTO variant confers a predisposition to obesity to be involved in the regulation of food intake rather than in the regulation of energy expenditure. As of the ten studies examining the influence of FTO variants, except for one based on questionnaires [5], all others reported an association with increased appetite whist three studies on energy expenditure showed no effects [4], [6], [8]. Despite this, the effects of global reduction in Fto function on energy homeostasis in mice are complex with *Fto* null and hypomorphic mice having low adipose stores with increased energy expenditure. Our findings demonstrate that reduction in Fto expression selectively in the ARC results in increased food intake, a finding consistent with the hyperphagia seen in mice lacking Fto and congruent with the fact that fasting (a strong stimulus to eating) reduces *Fto* mRNA levels in the ARC [16]. Over-expression of Fto in either ARC or PVN caused a reciprocal reduction in food intake. Our paradigm of stereotactic injection of AAV into the ARC or PVN does not permit correlations between physiological end points and levels of gene expression, because only a subset of the total neurons within each nuclei were infected. However, it is noteworthy that reduction in *Fto* expression in only a limited number of ARC cells is sufficient to affect food intake.

mRNA levels of *Agrp*, *Pomc* and *Npy*, ARC-expressed genes classically associated with the control of food intake, were not affected by the manipulation of Fto expression. However, over expression of Fto resulted in a 4-fold increase in the mRNA levels of *Stat3*, suggesting a possible candidate for the mediation of Fto's actions within the ARC. Stat3 is a ubiquitous transcription factor that is indispensable during early embryogenesis. In adult tissue, in addition to its critical role in leptin receptor signalling [19], Stat3 has also been shown to play crucial roles in a variety of biological functions including cell growth, apoptosis and cell motility [20]. The increase in Stat3 expression associated with overexpression of Fto and reduced food intake is consistent with its potential role in mediating leptin's anorectic effects. Tyrosine hydroxylase (TH) expression in the ARC was inversely modulated by Fto. It is tempting to speculate that Fto's effect on TH, may be relevant to the energy expenditure phenotype seen in *Fto* null mice by acting on the norepinephrein pathways or on the dopaminergic reward pathways for effects on food intake. However, the physiological relevance of these findings require further exploration.

In conclusion, our findings provide further support for the notion that FTO itself can influence key components of energy balance, most likely acting through central action on appetite, and is therefore a strong candidate for the mediation of the robust association between *FTO* intronic variants and adiposity. Importantly they provide the first indication that selective alteration of Fto levels in the hypothalamus can influence food intake, a finding consistent with the reported effects of *FTO* alleles on appetite and food intake in man.

## Materials and Methods

### Ethics Statement

All animal procedures were carried out in strict accordance with regulations and guidelines of the United Kingdom Home Office.

### Animals

Adult male Wister rats (250–300g, Charles River) were single housed throughout the study. All rats were maintained under controlled temperature (22°C) and on a 12-h light, 12-h dark schedule (lights on 7:00–19:00). Standard chow (Special Diet Services, SDS) and water were available *ad libitum*. For the high fat diet study, groups of male Wister around 200g were put on a 45% fat diet (D12451, SDS) for 10 weeks. Prior to all procedures animals were allowed to acclimatise for at least one week.

### Design and Construction of AAV Vectors

Six pairs of oligos of 19 nucleotides within the *Fto* coding region of the rat were designed using Ambion software and the efficacy of these sequences in knocking down the *Fto* transcript was tested in cultured GT1-7 cells. The two most effective pair of sequences were cloned into a hairpin structure. Briefly, the olionucleotide, containing either one of these 19 nucleotide sequences, separated by a short spacer from the reverse complement of the same sequence and a five thymidine termination signal, was cloned into the pSilencer expression vector system (Ambion) downstream of an U6 RNA polymerase III promoter. The U6 promoter, *Fto* hairpin sequence and terminator sequences were subcloned into the multicloning site of the pAAV-MCS backbone (Stratagene) to generate the corresponding AAV-shRNA vectors. An AAV vector expressing a scrambled shRNA sequence was used as a control for the knock-down study. To generate *Fto* expressing AAV vectors, the cDNA of murine *Fto* was cloned into an AAV backbone plasmid under the control of the ubiquitous hybrid promoter CAG that contains the chicken ß-actin promoter and CMV enhancer. Empty AAV vectors expressing no proteins were used as a control for the overexpression study. AAV-GFP was also produced to initially test for optimum serotype and titre, as well as accuracy of intranuclear injections.

### Viral Production and Purification

Vectors were generated by helper virus-free transfection of HEK293 cells using three plasmids [21] with modifications [22]. Cells were cultured to 70% confluence in roller bottles (RB) (Corning) in DMEM supplemented with 10% FBS and then co-transfected with: 1) a plasmid carrying the expression cassette flanked by the viral ITRs (described above); 2) a helper plasmid carrying the AAV *rep* and *cap* genes; and 3) a plasmid carrying the adenovirus helper functions. In this study we initially used plasmids carrying serotype 5,7,8,9 cap genes to generate AAV-2/5,2/7,2/8, and 2/9-GFPs to obtain optimal viral infection for the hypothalamic nuclei. GFP immunohistochemistry was performed 7 days after injections. AAV-2/7 seemed best for the hypothalamus, thus all the experimental virus were generate using serotype 7 cap genes. Plasmids carrying the adenovirus helper functions and cap genes were kindly provided by K.A. High, Children's Hospital of Philadelphia. Vectors were purified by two consecutives cesium chloride gradients using an optimized method [23], dialyzed against PBS, filtered, titred by qPCR and stored at −80°C until use.

### Stereotactic Surgery

Weight-matched rats were anesthetized with 40mg/kg ketamine and 20mg/kg xylazine, injected intramuscularly. Under anesthesia, a temporary 31 gauge needle (Hamilton) was inserted into the ARC or PVN according to the stereotactic coordinates of Paxinos and Watson [24]. The stereotactic coordinates for injection into the ARC were 2.8mm caudal to bregma, 0.4mm lateral to the midline and 10mm below the surface of the skull; for PVN 1.8mm caudal to bregma, 0.5mm lateral to the midline and 8mm below the surface of the skull. With a 10ul Hamilton micro-syringe, 1ul of 2×10^12^vg/ml vector preparations were injected into the nuclei over a 10min period. After vector delivery, the needle was left in place for 5 min to prevent reflux. Food intake and body weight were measured daily for two weeks following AAV microinjection, and were analyzed beginning on day 4 to allow time for postoperative recovery and for adenoviral gene to be expressed. At the end of the study, 3 weeks after AAV injection, whole brains were rapidly isolated and frozen on dry ice. Mesenteric and retroperitoneal fat pads were also dissected and weighted to the nearest 2 decimal place.

### Histological Analysis

For double-labelling of tissue sections with both isotopic and non-isotopic cRNA probes, a modification of our previously described protocol to detect *Fto* expression [16] was employed. Briefly, brains were sliced at 14um thickness and mounted on poly-L-lysine-coated slides (Fisher Scientific), dried overnight and then stored at −80°C. Slides were fixed in 4% paraformaldehyde followed by a microwave pretreatment with 10mM sodium citrate buffer, pH6.0 and dehydrated using a graded concentrations of ethanol (50–100%). ^35^S-labelled Fto cRNA was prepared as previously described [16], in addition, as a counterprobe, a digoxigenin (DIG)-labelled Pomc cRNA probe was prepared. Dig-labelled probes for sense and antisense *Pomc* mRNA were generated from the cDNA template corresponding to exon3 of *Pomc* in a pGem- T vector (Promega) and transcribed by SP6 or T7 RNA polymerase (Progema). ^35^S-labeled *Fto* probes and DIG-labelled *Pomc* probes were synthesized in parallel with either ^35^S-labeled UTP (GE Healthcare) or DIG-labelling mixture containing 10 mM ATP, CTP, GTP; 6.5 mM UTP; 3.5 mM DIG-labelled UTP (Boehringer Mannheim). Both probes were added to the hybridization buffer at the same concentration and sections were hybridized overnight at 57°C. After hybridization, slides were RNase treated followed by immunohistochemical detection of DIG. This was achieved by incubation of the brain section in blocking buffer containing 1∶500 dilution of AP conjugated anti-DIG antibody (Boehringer Mannheim) follow by the incubation in the detection buffer containing NBT, BCIP and levamisole. Slides were then covered with K5D emulsion (Ilford) and exposed for at least 2 weeks for the detection of ^35^S signal. Cells were considered positive for a message (for either DIG or ^35^S) if the signal was at least twice the background level. To assess differential sensitivity between isotopic and non-isotopic probes, the experiment was run with reversed labelled probe (i.e. Fto cRNA labelled with DIG and *Pomc* labelled with ^35^S).

To validate injection site and to establish a time course of protein expression for AAV2/7 serotype, at 3, 7 and 60 days after AAV-GFP injection, rats were anesthetized and perfused with 4% paraformaldehyde. Brains were fixed with 4% paraformaldehyde by transcardial perfusion and processed to produce 35um coronal cryostat sections. Successful injection and optimal virus titer were determined by the presence of GFP producing cells in the ARC and the PVN.

### RT-PCR

Micro-punches of ARC and PVN from individual rats were obtained based on the method originally described by Palkovits [25] with the shape of the basal hypothalamus, the ventricles and the major tracts serving as landmarks for localization during the process of removal. Accuracy of the micro-punches was confirmed with cresyl violet staining on the cryostat sections after nuclei removal. RNA was isolated from the micro-punch using the PARIS™ system (Ambion) and 200ng of purified RNA were used in a random-primed first strand cDNA synthesis reaction, using Superscript III reverse transcriptase (Invitrogen). Quantitative PCR reactions for *Fto* were performed in triplicate on an ABI 7900HT (Applied Biosystems) and using ABI PCR master mix, according to manufacturer's protocols. Arcuate expression of *Agrp*, *Pomc* and *Npy* was also quantified by real time PCR in these same samples, but the RNA had to be amplified before being used as template. Briefly, 5ng total RNA was DNase treated (TURBO DNase^TM^, Ambion) and subjected to the WT-OVATION™ Pico RNA Amplification System (NuGen Technologies, Inc) according to manufacturer's instructions. For the real time quantitative PCR data, all statistics were done on delta Ct values normalized to *B-actin* and expressed as ratio of control.

### Western Blot Analysis

Lysates for western blots were prepared using the PARIS™ kit with a protease inhibitor cocktail (Sigma). Western blots were probed with mouse monoclonal antibodies directed against the C-terminal FTO (1∶500; Abcam). B-actin (1∶1000; Abcam) was used as a loading control. Bands were visualized and semi-quantified using Chemoluminescence (Syngene).

### Statistical Analysis

All values are expressed as mean ±S.E.M. For each variable, statistical comparison between control and treated groups for each site were performed by two-tailed unpaired Student's t-test. For the analysis of food intake and body weight over time, two-way repeated measures ANOVA was used with time and treatment as variables for comparison. Statistical analysis was performed using Graph Pad Prism software (GraphPad Prism).

## References

[1] Coll AP, Farooqi IS, O'Rahilly S (2007). The hormonal control of food intake.. Cell.

[2] Frayling TM, Timpson NJ, Weedon MN, Zeggini E, Freathy RM (2007). A common variant in the FTO gene is associated with body mass index and predisposes to childhood and adult obesity.. Science.

[3] Loos RJ, Bouchard C (2008). FTO: the first gene contributing to common forms of human obesity.. Obes Rev.

[4] Cecil JE, Tavendale R, Watt P, Hetherington MM, Palmer CN (2008). An obesity-associated FTO gene variant and increased energy intake in children.. N Engl J Med.

[5] Hakanen M, Raitakari OT, Lehtimaki T, Peltonen N, Pahkala K (2009). FTO genotype is associated with body mass index after the age of seven years but not with energy intake or leisure-time physical activity.. J Clin Endocrinol Metab.

[6] Haupt A, Thamer C, Staiger H, Tschritter O, Kirchhoff K (2009). Variation in the FTO gene influences food intake but not energy expenditure.. Exp Clin Endocrinol Diabetes.

[7] Sonestedt E, Roos C, Gullberg B, Ericson U, Wirfalt E (2009). Fat and carbohydrate intake modify the association between genetic variation in the FTO genotype and obesity.. Am J Clin Nutr.

[8] Speakman JR, Rance KA, Johnstone AM (2008). Polymorphisms of the FTO gene are associated with variation in energy intake, but not energy expenditure.. Obesity (Silver Spring).

[9] Timpson NJ, Emmett PM, Frayling TM, Rogers I, Hattersley AT (2008). The fat mass- and obesity-associated locus and dietary intake in children.. Am J Clin Nutr.

[10] Wardle J, Carnell S, Haworth CM, Farooqi IS, O'Rahilly S (2008). Obesity associated genetic variation in FTO is associated with diminished satiety.. J Clin Endocrinol Metab.

[11] Wardle J, Llewellyn C, Sanderson S, Plomin R (2009). The FTO gene and measured food intake in children.. Int J Obes (Lond).

[12] den Hoed M, Westerterp-Plantenga MS, Bouwman FG, Mariman EC, Westerterp KR (2009). Postprandial responses in hunger and satiety are associated with the rs9939609 single nucleotide polymorphism in FTO.. Am J Clin Nutr.

[13] Tanofsky-Kraff M, Han JC, Anandalingam K, Shomaker LB, Columbo KM (2009). The FTO gene rs9939609 obesity-risk allele and loss of control over eating.. Am J Clin Nutr.

[14] Fischer J, Koch L, Emmerling C, Vierkotten J, Peters T (2009). Inactivation of the Fto gene protects from obesity.. Nature.

[15] Church C, Lee S, Bagg EA, McTaggart JS, Deacon R (2009). A mouse model for the metabolic effects of the human fat mass and obesity associated FTO gene.. PLoS Genet.

[16] Gerken T, Girard CA, Tung YC, Webby CJ, Saudek V (2007). The obesity-associated FTO gene encodes a 2-oxoglutarate-dependent nucleic acid demethylase.. Science.

[17] Boissel S, Reish O, Proulx K, Kawagoe-Takaki H, Sedgwick B (2009). Loss-of-function mutation in the dioxygenase-encoding FTO gene causes severe growth retardation and multiple malformations.. Am J Hum Genet.

[18] Meyre D, Proulx K, Kawagoe-Takaki H, Vatin V, Gutierrez-Aguilar R (2009). Prevalence of loss of function FTO mutations in lean and obese individuals.. Diabetes.

[19] Diabetes. Bjorbaek C, Uotani S, da Silva B, Flier JS (1997). Divergent signaling capacities of the long and short isoforms of the leptin receptor.. J Biol Chem.

[20] Akira S (2000). Roles of STAT3 defined by tissue-specific gene targeting.. Oncogene.

[21] Matsushita T, Elliger S, Elliger C, Podsakoff G, Villarreal L (1998). Adeno-associated virus vectors can be efficiently produced without helper virus.. Gene Ther.

[22] Wright JF, Le T, Prado J, Bahr-Davidson J, Smith PH (2005). Identification of factors that contribute to recombinant AAV2 particle aggregation and methods to prevent its occurrence during vector purification and formulation.. Mol Ther.

[23] Ayuso E, Mingozzi F, Montane J, Leon X, Anguela XM (2009). High AAV vector purity results in serotype-and tissue-independent enhancement of transuction efficiency.. Gene Ther.

[24] Paxinos G, Franklin KBJ (2007). The rat brain in stereotaxic coordinates, 6th Edition.

[25] Palkovits M (1973). Isolated removal of hypothalamic or other brain nuclei of the rat.. Brain Res.

